# Implementing individual placement and support (IPS): the experiences of employment specialists in the early implementation phase of IPS in Northern Norway. The IPSNOR study

**DOI:** 10.1186/s12888-021-03644-x

**Published:** 2021-12-20

**Authors:** Cathrine Moe, Beate Brinchmann, Line Rasmussen, Oda Lekve Brandseth, David McDaid, Eóin Killackey, Miles Rinaldi, Marit Borg, Arnstein Mykletun

**Affiliations:** 1 Nordland Hospital Trust, Centre for Work and Mental Health, Bodø, Norway; 2 Faculty of Nursing and Health Sciences, Nord University, Bodø, Norway; 3 Department of Community Medicine, UiT – The Arctic University of Norway, Tromsø, Norway; 4 Care Policy and Evaluation Centre, Department of Health Policy, London School of Economics and Political Science, London, UK; 5 Orygen, Melbourne, Australia; 6 Centre for Youth Mental Health, The University of Melbourne, Melbourne, Australia; 7 South West London and St George’s Mental Health NHS Trust, London, UK; 8 University of South-Eastern Norway, Drammen, Norway; 9 Centre for Research and Education in Forensic Psychiatry, Haukeland University Hospital, Bergen, Norway; 10 Division for Health Sciences, Norwegian Institute of Public Health, Oslo, Norway

**Keywords:** Early phase, Employment specialist, Implementation, Individual placement and support, Mental illness, Rural, Supported employment, Vocational rehabilitation, Work

## Abstract

**Background:**

For decades there has been a continuous increase in the number of people receiving welfare benefits for being outside the work force due to mental illness. There is sufficient evidence for the efficacy of Individual Placement and Support (IPS) for gaining and maintaining competitive employment. Yet, IPS is still not implemented as routine practice in public community mental health services. Knowledge about implementation challenges as experienced by the practitioners is limited. This study seeks to explore the experiences of the front-line workers, known as employment specialists, in the early implementation phase.

**Methods:**

Qualitative data were collected through field notes and five focus group interviews. The study participants were 45 IPS employment specialists located at 14 different sites in Northern Norway. Transcripts and field notes were analysed by thematic analyses.

**Results:**

While employment specialists are key to the implementation process, implementing IPS requires more than creating and filling the role of the employment specialist. It requires adjustments in multiple organisations. The new employment specialist then is a pioneer of service development. Some employment specialists found this a difficult challenge, and one that did not correspond to their expectations going into this role. Others appreciated the pioneering role. IPS implementation also challenged the delegation of roles and responsibilities between sectors, and related legal frameworks related to confidentiality and access. The facilitating role of human relationships emphasised the importance of social support which is an important factor in a healthy work environment. Rural areas with long distances and close- knit societies may cause challenges for implementation.

**Conclusion:**

The study provides increased understanding on what happens in the early implementation phase of IPS from the employment specialists’ perspective. Results from this study can contribute to increased focus on job satisfaction, turnover and recruitment of employment specialists, factors which have previously been shown to influence the success of IPS. The greatest challenge for making “IPS efficacy in trials” become “IPS effectiveness in the real world” is implementation, and this study has highlighted some of the implementation issues.

## Background

The number of people with severe mental illness excluded from the work force has been rising for years in developed countries [1]. Employment is shown to enhance social inclusion and self-esteem [2], and appropriate work can be essential for recovery from mental illness [3]. Individual placement and support (IPS) is an evidence based approach for helping people with severe mental illness to gain and maintain competitive employment [4]. One of the purposes of IPS is to challenge the premise that people with serious mental illness cannot work [5]. The efficacy of IPS is reported in three Cochrane reviews [6–8] and meta-analyses covering 21 different randomised controlled trials across Europe, Asia and North America [9, 10]. A new meta-regression study demonstrated that IPS efficacy was surprisingly robust. The study found negligible effect modification by country, welfare system and labour market situations [11]. Yet, the challenge of sustainability in mainstream practice beyond trials is a well-known phenomenon [12–14] and we do not know as much about what happens in mainstream practice in the process of implementing IPS in a non-trial environment. Also, the implementation of IPS may be more country and culture specific than the efficacy of IPS. For this study, implementation is a “specified set of activities designed to put into practice an activity or program of known dimensions” ([15] , p.5). Most studies of IPS implementation are retrospective, summarising the process after a period of operating time for what is seen as a project rather than an established service [16]. The major challenges concerning IPS implementation within the context of a project are a lack of stable funding after the project period is over, limited supervision and high turnover of employment specialists, inadequate interagency collaboration, inadequate integration into mental health services, adverse clinician and societal attitudes and cultures, as well as organisational barriers [17–21].

The implementation process for IPS can be challenging as it is dependent on several actors and agencies for success. Various stakeholders such as clinicians and public unemployment agency staff are actors in the IPS implementation process [17, 22]. The present study aims to explore the experiences of the front-line workers, known as employment specialists, during the early implementation process. Employment specialists, together with their supervisors, “enact the IPS principles” and are therefore core actors in the implementation process [23]. The employment specialists’ key role is to support clients in finding and retaining a meaningful job in a competitive working environment by using the IPS approach. Employment specialists address clients’ vocational needs and ensure that vocational goals are given high priority. The employment specialist role includes tailored collaboration with job seekers, clinicians, public unemployment agency advisors and employers, and a key function for the employment specialist is to coordinate vocational plans with all actors [23]. The employment specialists also need to develop a good knowledge of local labour markets and employment opportunities [24, 25]. IPS supervisors are responsible for training, supervision and evaluation of employment specialists.

Previous studies have investigated the preferred competencies for employment specialists [26, 27] and employment specialists’ view of facilitators and barriers to employment for individuals experiencing mental illness [28, 29]. A recently published review of experiences of participating in IPS found that the employment specialists see themselves as culture brokers between mental health services and the business world [30]. Bonfils’ [31] recent study focuses on the integration of employment specialists into Danish health teams from the health managers point of view. Their study shows how IPS is regarded as a parallel rather than an integrated service, and as a supplement to treatment rather than a mutual responsibility. Our study adds to the knowledge base on the employment specialist role, by gaining new knowledge on their experiences within the early phase of implementing IPS. Tansella and Thornicroft [32] describe early implementation as the phase where someone has “decided in principle to use evidence based practice to shape routine clinical care” p.283.

Implementation of IPS requires policy accommodations to the health and labour services at a local, as well as governmental level [19]. Our study took place in a Norwegian context where national policy mandates widespread implementation of IPS [33, 34] and IPS has systematically been studied in Norwegian trials since 2012 [35]. Norway is a high-income society, characterised by a generous welfare system and a low general unemployment rate [36]. Norway also has the highest sickness absence rate in the world, and is among countries with the highest levels of disability and rehabilitation benefits [37]. Health and social services are rooted in two different sectors, regulated through different legislation and funded separately. The Norwegian mental health services provide community- based and specialised, hospital care [35]. For health services, local provision is influenced by governmental authorities through legislation, but local authorities are free to organise and arrange services to meet local conditions [38]. The Norwegian Labour and Welfare Administration (NAV) provides social and vocational services as well as social welfare benefits. NAV offices represent Norway’s Public Employment Services, defined at EU level as “the authorities that connect jobseekers with employers” ([39], para 1). Two directorates, the Health Directorate and the Labour- and Welfare directorate, are involved in the implementation of IPS nationally.

The purpose of this study was to explore and describe employment specialists’ experiences of the early phase of IPS implementation in a non-trial context. A broad approach to the research field was conducted to embrace a range of barriers and facilitators in the implementation process. The study intends to increase knowledge of what happens in the practice field after the decision to implement IPS is taken, from the employment specialists’ point of view.

## Methods

This qualitative study was carried out by a research team advised by practitioners from the subject area (employment specialists and educators of employment specialists) and a person with lived experience of mental illness who has accessed an IPS program. The research participants in this team were responsible for creating the various stages of the research process, including discussions of methods of data collection, interview-guide development, recruiting, analysis and dissemination. Practitioner and service-user involvement in health and social research has gradually been given more priority in research policies, both in Norway and internationally [40].

### Contextual background for the study

The context of this study is a scale-up of IPS services in Northern Norway. Northern Norway consist of two counties (Nordland, and Troms and Finnmark), 87 municipalities and a total population of 463,000 with a density of 4.1 person per km^2^. There are only two towns with more than 50,000 inhabitants, Bodø and Tromsø and the region is characterised by rural areas and long distances between towns and smaller settlements. The process of establishing IPS in northern Norway started with Bodø in 2013. Over the last 2 years, the IPS services have expanded from four to 14 sites. Currently, 45 full-time employment specialists are employed across these 14 sites. The population coverage is approximately one employment specialist per 10,000 inhabitants in the general population. Most IPS teams consist of two or three employment specialists who are typically seated in different health teams.

The present study is a part of IPSNOR [41] which is a research and implementation project at the Centre for Work and Mental Health (KAPH) at Nordland Hospital trust. KAPH has encouraged and helped sites to apply for funding to establish IPS, write collaboration agreements and to develop networks. KAPH has also offered implementation support for IPS actors in Northern-Norway. This support includes training, supervision and a two-day secondment stay (job shadowing) for the employment specialists. Lectures have also been offered to health and NAV personnel at each site. All IPS services in Northern Norway are part of the IPSNOR network, led by KAPH. The IPSNOR network is inspired by the IPS Learning community [42]. IPSNOR also has a role in organising and conducting fidelity reviews. IPSNOR research aims to gain knowledge about how the implementation is happening, and to learn about the consequences for society at large and for the individuals.

### Study participants and data collection

#### Participants

Study participants were 45 IPS employment specialists located in Northern Norway. Among the 45, 11 were IPS supervisors who typically had a dual role as an employment specialist and a supervisor. This meant that all 14 IPS sites were represented by employment specialists and supervisors. Nearly all participants worked in newly established IPS teams as most of these IPS sites were established in 2019. Although some sites had been running for a while, turnover of employment specialists and expansion of teams meant that, at the time of data collection, most participants had only been employed within the previous year. Most participants were employed by NAV and had their daily work embedded within a mental health team. The mental health team could either be within a community or hospital outpatient health service. The employment specialists had various professional backgrounds. Some had health or social care qualifications, like nurses and social workers, while others had work experience from insurance, music or artisan industries. Their ages ranged from 25 to 55. There were approximately 25 female and 20 male participants; the number varied due to turnover during research process. For the interviews there were 21 women and 12 men. The participants of the study are hereafter referred to as employment specialists.

#### Data collection and data development

Data for this study consists of 180 pages of fieldnotes and transcripts from focus group-interviews.

Fieldnotes*:* author CM participated as an observer at various settings for education, training and supervision of employment specialists as per the method of Fangen [43]. Fieldnotes were taken during these observations, trying to capture the early experiences of the employment specialists. The fieldwork for meetings in which employment specialists were represented began in February 2018. For the first time, in September 2019, all the employment specialists from Northern Norway took part in a two-day seminar. As part of this seminar, employment specialists were divided into eight groups, and shared their early practice experiences with each other. A researcher or advisor from the IPSNOR network took fieldnotes from some of the groups and the following plenary discussions, led by authors AM and BB, and advisors LHH and MJ.

Focus group interviews: The second two-day seminar for all employment specialists in Northern Norway took place in January 2020. The theme of this seminar was about challenges in the implementation of IPS and strategies to meet these challenges. The topic was chosen based on dialogue with employment specialists in September 2019. Thirty-three employment specialists participated in this seminar. Five focus group interviews were conducted with the 33 employment specialists, following the method of Krueger and Casey [44]. The moderators of the groups were researchers from the IPSNOR research team. An interview guide was prepared based on notes from the September seminar, and adjusted after comments from the employment specialist advising the research (MJ). The participants were encouraged to speak openly about their work experiences with IPS. After the focus group interviews, the five moderators of the groups met and shared their immediate reflections. These were written and laid the foundations for further analyses. The written notes were also the basis for a plenary discussion after the interviews (led by authors MR and EK). The interviews were audio recorded and lasted for 90 min.

### Data analysis

Throughout the research process we have noted and searched for patterns of meanings and issues of potential interests in the data. The field notes from participant observations and the first seminar were carefully read and systematised (by author CM and advisor AS) following Braun and Clarke’s method of thematic analysis [45]. The themes from this first analysis laid the foundations for the development of the focus group interviews and the interview guide. The focus group interviews were transcribed verbatim. The transcripts were carefully read and coded relating to the scope of the study. Authors LR, OLB, MB and CM coded the transcriptions. We systematised the codes in themes and subthemes, and these were presented to author BB and advisor LHH in the research team. Comments were noted and included in the further analysis process. The analysis has involved a constant iterative back and forth process, moving between the entire data, the codes and the produced analysis [45]. Table 1 shows an example of codes, subthemes and themes from the analysis process. The interviews and analysis were conducted in Norwegian. Quotes were translated to English in the last analytic and writing phases.

**Table 1:** Example of codes, subthemes and theme

Theme	Subthemes	Codes	Transcribed text
Pioneers of service development	Unclear who leads the IPS implementation	Someone else should do the implementation work.	We wish we could call IPSNOR so they could come and do the [implementation] work for us.
		Expected more to be ready before start.	Some things should have been ready before they applied for employment specialists. The advertisement did not say anything about leading the implementation.
		The managers should join IPS seminars. They are the decision makers.	If we shall find solutions, it is a problem that we don’t have our leaders at these seminars because I don’t make decisions.
	IPS services need to be developed at each site based on local capacities and conditions.	Want directives on how to establish IPS.	I want directives on how to do this [the implementation] so I can stop spending time and energy on getting new routines all the time.
		Work different based on where they are employed.	There are differences on how we work based on who is paying our salary. It should not be like this. We are being fidelity reviewed based on the same criteria, but our prerequisites are different.
		Wish for national guidelines on documentation in patient records.	It should be decided from the departments: everyone that works with IPS should have access to these tools and documents.
		Local differences	We live in a small community. We cannot work in the same ways as in big cities.

## Results

Four central themes were derived from the analysis process: 1. The employment specialists are pioneers of service development. 2. There are unsettled partnerships between people and sectors. 3. There are particular challenges in implementing IPS in small communities and rural areas. 4. The role of human relationships in facilitating IPS implementation. These themes comprise several subthemes and are presented below.

### Pioneers of service development

The data analysis revealed that the early phase of implementation involved IPS service development. The employment specialists described how IPS services needed to be developed at each site based on local capacities and conditions. Some of the employment specialists described that when they started, they were not prepared for all the implementation work that had to be done. They had expected someone else to lead the implementation, and that fundamental structures would be in place beforehand. They missed decisions and organisational structures such as who was deemed responsible for the IPS service locally, which health teams should they belong to, where should their work desks be located, and what kinds of information and monitoring systems should be available. They also missed decisions about determining not only to whom they should report, but also what information should be presented. As many of these issues were unclear and not sorted before the employment specialists started, they had to spend time on service development, sometimes in parallel with their work with clients. Some employment specialists found this pioneering work exciting and enjoyed the process of shaping their own job situation, while others felt that they did not have the necessary capacities, knowledge or skills to do such implementation work: “We are lay people, supposed to change routines in two massive systems where well-educated people work” [well-educated within health and welfare].

Some employment specialists found it surprising that each site was supposed to develop ways to implement the IPS service. They would have liked to have seen a national management policy on how IPS is implemented and delivered in Norway. They were aware of overarching national strategies, but local decisions related to implementation remained. The participants also requested more involvement from managers of the health and welfare services. Although formal collaborative agreements were signed and formalities were in place before IPS start-up, it seemed unclear to some of the employment specialists as to who had the local responsibility for, and ownership of, the implementation of IPS. The study revealed that a proactive leader with engagement and knowledge of IPS was of great benefit in the early phase. Some employment specialists explained how managers actively engaged in challenges as they emerged and thus solved practical issues quickly: “I know about one municipality where the manager has a great belief in IPS. She facilitates an interdisciplinary team and everyone ‘burns for’ getting people into work. They start thinking about work immediately, and everyone is engaged”. Other employment specialists explained how they felt lonely and not supported by their managers because of what they experienced as lack of involvement in the implementation of IPS and absence of IPS knowledge. They wished for IPS education for their managers in addition to the KAPH education:

I think there should be a requirement for IPS education for managers when applying for funding. We tried to get our managers [to come] with us to the national IPS courses so they could learn as well. Now, we need to come back to our teams and explain how things should be done. I don’t think it is our job.

Some employment specialists were pushy and expected others to be involved, while other employment specialists did not want to be a burden to busy health and NAV professionals: “It is fragile. We need to maintain a continuous pressure to create changes. Not all of us can manage that”.

The employment specialists said that the strategies for the IPS service development were to find the key people and build relationships with them. These key people differed from site to site. Implementing the IPS service was continuous work, and the participants revealed that being enthusiastic was not enough; “actors from the whole system have to engage”. Two IPS fidelity scales exist to measure program fidelity and validity [46, 47] . The scale items provide concrete indications that practice is being implemented as intended, and the fidelity of each IPSNOR site is reviewed within the first year. Some employment specialists did not feel they had enough authority to be listened to in their clinical team, and they looked forward to the fidelity review with the hope that the fidelity report would strengthen their authority in order to be heard by local stakeholders.

### Unsettled partnerships between people and sectors

A prerequisite for the IPS service was close collaboration between health services and NAV. For the employment specialists, trying to manoeuvre several sectors and actors, there were challenges both concerning organisational and cultural factors. For the employment specialists starting at a newly created service, like a flexible assertive community treatment- team, it was easier to implement IPS. The whole team was established together from the start, and the employment specialists were integrated within an interdisciplinary team. To create new collaborative structures in established services was more challenging. How this was supposed to be done was unclear from the employment specialists’ point of view as they were not familiar with the original structures. One employment specialist explained how he actively worked on the partnerships between sectors: “I realised early on that I did not come to a settled table. I needed to do the work on building relationships to different teams and employers quite actively”. Most of the employment specialists felt welcome at the health teams, but still many of them did not consider themselves integrated: “We are well integrated in many ways. We are invited to Christmas parties and everything. But when it comes to patients and confidentiality – it stops.”

Issues that were highlighted as challenging in implementing IPS between sectors were:

#### Balancing the presence in health and welfare offices and the labour market with activity registration and practice documentation

Most of the employment specialists had their daily work seated within a health team. As they were employed at NAV, they were also expected to be present at meetings in the NAV office and to document activities in the NAV documentation system. According to the IPS manual [48] and IPSNOR routines, employment specialists reported on a number of variables to their supervisors weekly. During interviews, the employment specialists told how they strove to balance their time spent in the NAV office, the health team and out in the “workplace and client’s daily life” with activity registration claims and practice documentation: “I have to please so many; NAV, the clinicians, employers etcetera. I am a team-player but I do not feel I have a team. I feel alone”.

#### Ambiguity on confidentiality accessing two sectors

Although the employment specialists are embedded in health teams, as most are employed at NAV, they do not have access to patient information held by the health teams due to confidentiality legislation. The employment specialists stated that rules on patient confidentiality were implemented differently by various health teams and sites. As some of the employment specialists did not have access to patient information held by the health services, they were not allowed to participate in clinical meetings. In some teams however, managers found solutions. For instance, some employment specialists had a 0% working contract at the health service or the clinicians de-identified patients before presenting them to the team. Some employment specialists did not have access rights to their clients’ medical records and that made them dependent on others to ensure that IPS activities were documented in the clinical record. As most of the employment specialists were employed by NAV, they had full access rights to welfare system data. For the minority of employment specialists employed in a health service, IPS information was documented through the clients’ NAV advisor.

#### Handling information in a complex setting

The employment specialists needed to balance the delivery of what was quite complex information through different sectors. They sometimes found it hard to know what to write in the welfare documentation system to avoid breaking the confidentiality rules of the health sector. Employment specialists were seen as coordinators between services and with joint contracts they had to handle different and complex laws and regulations.

#### Knowledge influences partnerships

Most employment specialists described having received sufficient education and training to work with IPS. However, they perceived a lack of IPS knowledge in practitioners and managers in the health and NAV services, leading to a lack of understanding of the importance of close collaboration between sectors and the integration of employment specialists in the clinical team: “I believe our integration and the IPS practice depends on the knowledge and beliefs of the managers”. Also, the employment specialists noted that some clinicians made a distinction between treatment and work-related issues. They also revealed that both clinicians and NAV advisors were restrictive in referring patients with moderate and severe mental illness to IPS.. The employment specialists noted that with increased knowledge, the perspectives of the health and NAV professionals changed, and the most effective source of knowledge was their own successful narratives. During interviews, employment specialists who had worked for a while were more optimistic concerning the implementation of IPS than new employment specialists. “It will be better, it just takes some time”.

### Particular challenges in implementing IPS in small communities and rural areas

Most of the IPS teams were located in small communities in rural areas. This involved challenges that were different from implementing the service in urban areas. Issues related to small communities and rural areas include:

#### Organising the work to meet local needs

The IPS services’ in this study could serve areas approximately 1 h drive from the health team, the NAV office, a relevant employer and the client’s home. The employment specialists had to organise their working weeks so that they did not spend all their time driving between meetings. Therefore, they could not be as flexible on face-to-face meetings at short notice as their colleagues in urban areas: “We live in a small community. We cannot work in the same ways as in big cities”. Even though the employment specialists kept contact with collaborative actors through digital channels, they still spent a lot of time in their cars. They also had to work out of their cars as offices, making them have less contact with colleagues within the health and NAV offices. The job opportunities for clients were also influenced by long distances and small communities: “In our IPS service, two of the municipalities are small and there are not many job opportunities. A majority of the job seekers do not have a driver’s license and there are also limitations because of that.”

#### Pros and cons of local knowledge

Employment specialists had a good overview of their local communities and the local labour market, and knew several employers personally. This led to a low threshold for contact, which was good for IPS. The employment specialists also knew the skills and competencies sought by businesses, benefiting the process of finding a good job match between employer and client. Employment specialists stated that a barrier in the job seeking process in small communities was if a client had a past with, for instance, illegal drugs or threatening behaviour. This could make employers and work colleagues scared, and employment specialists needed to be aware of existing ongoing narratives in the community. The employment specialists said it could be hard to protect the job seeker’s confidentiality when they met outside of the office. Residents in small communities often know where people work, and others will recognise a person as a client if seen with an employment specialist during daytime. This led to a conflict between the need to spend time outside the office and finding a suitable place to meet clients outside of the office to protect their confidentiality.

In smaller communities, several residents may have different roles and the line between the professional and private might be unclear. This could influence the implementation of IPS. “I have met quite many clients, and I have had previous knowledge of everyone”. Employment specialists told stories during interviews of how their own partners also were potential employers for a client and how this would influence the job-seeking process both in a positive and negative way.

### The role of human relationships in facilitating IPS implementation

Most IPS teams consisted of two or three employment specialists, often seated in different health teams so they did not meet daily. Most IPS supervisors stated they had to shoulder responsibilities for implementation and personal support which they felt belonged to health and NAV managers. The supervisors had a good overview of the employment specialists’ work situation and they were afraid that their employment specialists would burn out and quit because of the unclear work situation.

Being part of the IPSNOR network allowed employment specialists and the supervisors to know about other employment specialists in similar circumstances. They could share experiences and learn from each other. When participating at employment specialist seminars, the employment specialists described having an opportunity to withdraw from their daily practice, get an overview over the field of knowledge and gain a reminder of why they were doing this. They felt seen and heard, and part of a bigger community:

“When working at home, we sometimes get stuck in local problems and the focus gets quite narrow. Here we see IPS in a bigger perspective and can see that IPS has a greater meaning. Having both national and international focus – it gets really big. I get really excited”.

Despite all the IPS implementation work, which disrupted the work with clients, the employment specialists remained enthusiastic concerning the development of IPS. Even if the implementation process took time and was left to the employment specialists to steer more than they expected, they gave the impression of experiencing their work to be meaningful and important. Most of the employment specialists had no prior experience of working with people living with severe mental illness and they needed to train on how to balance between a friendly push and respect for their client’s symptoms and the challenges they were facing. They often needed to understand and deal with their client’s ambivalence concerning work and sometimes their periodic withdrawal. Nonetheless, the work with clients was considered meaningful not burdensome. The pleasure of seeing a person get into work made the hard implementation work worth it: “It is worth it. It is so worth it when you get a job for a person”.

The employment specialists believed the support they received from the clinical teams was good. They received supervision on how to create relationships with people living with mental illness, and they received follow-up support after serious incidents like suicide attempts. They also received supervision from clinicians on how to maintain their client’s hope when a person is feeling hopeless. Collaboration and communication with employers were considered good from the employment specialists’ point of view. They found employers accommodating and socially responsible. The employment specialists shared examples of good teamwork with actors from different sectors, making them believe that implementation of IPS was possible to achieve. They also experienced a sense of coping when they handled chaos, resistance and uncertainty, giving them the courage to continue with what they perceived to be important implementation work.

## Discussion

The purpose of this study was to explore and describe employment specialists’ experiences of the early phase of IPS implementation in a non-trial context. The efficacy of IPS is well documented and there seems to be agreement that the evidence base for the efficacy of IPS is plentiful [9–11]. The issues for implementation then become more relevant. The model of IPS is well described, including the employment specialist’s role and work with clients [23, 28, 29] . This study contributes to the field of IPS implementation by providing knowledge of what happens in the practice field after the decision to implement IPS is taken.

Implementation is to put a program of known dimensions into practice [15]. To improve the outcomes of a service, Fixen et al. [49] stated there need to be a combination of effective programs and effective implementation methods. IPS can be seen as a quite simple service model, clearly described in manuals and research articles [48]. Despite having sufficient training, collaboration agreements and IPSNOR implementation support, the employment specialists experienced the implementation process to be complex and not straightforward. This supports findings from other studies reporting that IPS implementation can be challenging due to the involvement of multiple agencies, organisations and actors [50]. Our study shows how the employment specialists have significant roles in the implementation process and their role as pioneers of service development in the early implementation phase, from their perspective, was not sufficiently recognised and communicated to them before they started their jobs. Even though some of the employment specialists enjoyed this aspect of their work, the majority were surprised that they had implementation responsibility. To be effective in the early implementation phase, the employment specialists would have benefited from having competencies as pioneers of service development. This adds to the employment specialist competencies and skills identified in previous studies such as being collegiate, supportive, outreaching and team oriented [26, 27].

The employment specialists in this study emphasised the facilitating effect of human relationships in the implementation process. While being part of the IPSNOR network made the employment specialists felt seen, heard and supported, some reported feeling lonely and insufficiently supported locally. They had to shoulder the burden of much of the implementation work. Some employment specialists said they had to face responsibilities beyond what they felt capable of doing. The stakeholders’ level of ownership of IPS and collaboration have previously been identified as facilitators of IPS implementation [22], and the lack of support locally can relate to a lack of ownership of the local stakeholders. There was no clear mechanism to decide on who had the local responsibility for implementation. As identified by Moen et al. [30], the employment specialists see themselves as culture brokers between sectors. This current study supports their finding and further highlights the importance of good collaboration and clear allocation of responsibility. As no sector or person was obliged to lead implementation, the employment specialists and their supervisors found themselves taking on additional responsibilities over and above what they thought was in their job descriptions. Like Gilburt et al. [51] we therefore found that the focus was primarily on new service delivery rather than the adoption of a new way of approaching healthcare. The latter turned out to be important for the implementation in the clinical teams.

Both the identified competencies of the employment specialists and the facilitating effect of human relationships are elements affecting a healthy work environment [52, 53] . First, not being prepared for the development of implementation tasks, including a lack of expected competencies needed to be an implementation pioneer, can lead to job strain due to high job demands, low job control and role stress as identified in the systematic review by Harvey et al. [53]. Second, human relationships have a key impact on facilitation; this is fundamentally about having social support which is a crucial aspect of a healthy environment. High job strain and low social support in the workplace can lead to a risk of developing common mental health problems [53]. The supervisors in this study were afraid that the employment specialists would burn out and quit their jobs. High turnover of employment specialists is regarded as a one of the critical barriers to IPS implementation [17] and high job strain and lack of social support can be one reason why employment specialists quit their jobs. Rurality and the necessity for long distance car travel are an extra dimension of implementation work seen in the IPSNOR study. Even though rurality does not seem to influence the effectiveness of IPS [54], employment specialists, through necessity, might have to work more independently than would be the case in more urban settings. They carry out a lot of administrative work from their cars and strive to balance different expectations of being present in their offices with the need to engage in activity on behalf of their clients. The unclear line between private and professional life in a rural area is not an IPS specific issue, but it can affect implementation, as the interaction between these factors needs to be taken into consideration. Local conditions and contexts will vary between sites and employment specialists sometimes therefore need to balance their obligation to be loyal to the IPS model with the need to adapt the model to local circumstances. This is also reported in a Canadian study revealing that the shaping of supported employment services is affected by institutional pressures, employment specialist interactions and relationships with others, their beliefs, values and ideologies [55].

These findings also reveal how the structures and “ground” for implementation were unclear for the employment specialists. Even though the sites made a commitment to providing good quality IPS when applying for funding, this study reveals that neither the organisations nor the employment specialists were sufficiently well prepared for the complexity of implementing IPS. The responsibilities for implementation between sectors were unclear. Previous research suggests that the sectoral responsibility for IPS must be addressed more clearly as the service sits at the interface between public sectors responsible for welfare services and health services, which may confuse issues of ownership and responsibility [11]. Activities carried out prior to the start of the practical work, such as planning and fostering supportive organisational conditions, are especially important to the success of implementation, with a need for a mandatory service plan for coordinated implementation to be produced [56, 57]. The importance of involving actors besides the employment specialists, such as clinical teams, in implementation is highlighted in the IPS international literature. A shared ownership of IPS is needed to achieve service sustainability. The importance of offering training to all involved actors is highlighted [58, 59]. IPSNOR and national courses offer IPS training to a broad group of actors. Nonetheless, findings from this study show that local actors can be hard to engage in such training. The employment specialists hoped the fidelity reviews could strengthen their authority in reporting deficiencies in IPS practice. The role of high fidelity in the implementation is well described [58]. The planned fidelity reviews can therefore be beneficial to the experiences of the employment specialist.

Our study has important limitations. Since the participants of this study were brought together in seminars, we could not link their experiences to their contextual work situation. Therefore, we are not able to say if organisational or contextual factors facilitated or hampered implementation. The study therefore provides overarching themes for all employment specialists working in Northern Norway. Moreover, it should be acknowledged that when KAPH arrange seminars for employment specialist for education and data collection, this also affect the implementation process by facilitating social relations between different stakeholders as well as providing additional opportunities for social support. Therefore, one consequence of the research process itself has been the resolution of some of the implementation barriers. We believe the challenges of implementing IPS would have been even greater without this research process. It is also possible that the employment specialists highlighted barriers in the implementation process because they hoped that involvement from the research team could help resolve some of their issues as several of the researchers are closely connected to the practice field. Despite these limitations, the results of this study provide an increased understanding of, and shed light on, complexities in the early phase implementation of IPS from the perspective of “front line workers”, the employment specialists.

### Implications for practice and research

Findings from this study are relevant more broadly to implementation studies of complex interventions, and have implications for practice and research on IPS implementation. Critically, the role of the employment specialists as implementors needs to be the focus of much more attention when planning for implementation of IPS. The employment specialists can be better prepared for this implementation work before they become active at a newly established service. By communicating the pioneering nature of their role in developing IPS collaborative structures, the employments specialists can be better prepared. This has implications for the recruitment and induction of  employment specialists as well as job satisfaction and job retention. In the early phase, IPS education could put more emphasis on learning about the process of effective implementation, including collaboration building across sectors, in addition to learning about the practice of IPS. Some fundamental structures should also be in place before the employment specialists enter the field, such as ‘health team belonging ‘, that is being seen fully as being part of the health team. There is a need for standardised reporting routines and clarification of lines of accountability on who holds responsibility for IPS implementation. There is also a recommendation for a “start-up-plan” between all actors to coordinate implementation efforts. The facilitating effects of good human relationships emphasizes the need for social support within complex implementation work. The burden of responsibility for IPS implementation can be shared between the managers of the health and welfare services. It is therefore important that collaborative agreements are not only anchored at the top management level, but also reach managers closest to the employment specialists and the IPS practice. For IPSNOR, one implication of this study is the acknowledgement of the need to find ways to make local actors engage in the implementation. The documentation of implementation processes and valuable experiences can give institutions important knowledge for future implementation processes. We therefore see the value for IPS services to routinely record things that have helped make IPS implementation work well as well as things that have not worked well. In the light of pandemic and global environmental challenges, the use of technical solutions for encounters have increased. A normalisation of non face-to-face meetings can help the implementation of IPS in rural communities. It allows the employment specialists to have more frequent contact with network and job seekers without spending time traveling.

There are still knowledge gaps concerning other aspects of the implementation process of IPS, and we suggest more research exploring the process from the clinicians, welfare practitioners, employers and clients’ point of view. Future research should also focus on contextual factors facilitating or hampering the implementation process and the role of other actors in the implementation process. It is also important to explore ways to support the employment specialists during IPS implementation.

## Conclusion

This qualitative study provides increased understanding on what happens in the early implementation phase of IPS from the employment specialists’ point of view. The study shows how employment specialists are key to the implementation process. Implementing IPS entails adjustments in multiple organisations, which is more than filling a pre-defined role. Thus, we call this being pioneers of service development. Some found this as a difficult challenge not corresponding to their expectations going into this role, whereas others appreciated the pioneering role. IPS implementation also challenged the delegation of roles and responsibilities between sectors, and related legal frameworks related to confidentiality and access. The facilitating role of human relationships emphasised the importance of social support which is an important factor in a healthy work environment. Rural areas with long distances and close- knit societies may cause challenges for implementation. Both work strain and received social support can influence how the employment specialists experience their work to be healthy. The findings from this study can contribute to increased focus on job satisfaction and turnover of employment specialists which have previously been shown to influence the success of IPS. The greatest challenge for making “IPS efficacy in trials” become “IPS effectiveness in the real world” is implementation, and this study has highlighted some of the implementation issues.

## Data Availability

The datasets generated and analysed during the current study are not publicly available due to participant confidentiality but is available from the corresponding author on reasonable request.

## Abbreviations

IPS: Individual placement and support
KAPH: Centre for work and mental health
NAV: The Norwegian Labour and Welfare Administration
